# Assessment of Lipid Profile in Patients With Pulmonary Tuberculosis: An Observational Study

**DOI:** 10.7759/cureus.39244

**Published:** 2023-05-19

**Authors:** Anbumaran Parivakkam Mani, Shanmugapriya K, Deepak Kanna K, Sankalp Yadav

**Affiliations:** 1 Respiratory Medicine, Saveetha Medical College and Hospital, Chennai, IND; 2 Respiratory Medicine, Sri Lalithambigai Medical College and Hospital, Dr. M.G.R. Educational and Research Institute, Chennai, IND; 3 Respiratory Medicine, Tamil Nadu Government Multisuperspeciality Hospital, Chennai, IND; 4 Medicine, Shri Madan Lal Khurana Chest Clinic, New Delhi, IND

**Keywords:** ldl cholesterol, hdl-cholesterol, serum triglycerides, serum lipid profile, tuberculosis

## Abstract

Background: *Mycobacterium tuberculosis* causes tuberculosis (TB), an infectious lung disease. There is mounting evidence linking low lipid levels to a variety of human diseases, including TB. Cholesterol, mainly due to its involvement in heart disease, gets more attention in recent years. The objectives of the study were to look into the link that connects hypolipidemia to the existence of pulmonary/extrapulmonary TB; we have tried to find the link in relation to patients who have been recently diagnosed with TB as well as in those who are having TB in the long term.

Materials and methods: An observational study was performed on TB patients attending respiratory medicine at the Saveetha Medical College and Hospital, Chennai, Tamil Nadu, India, from February 2021 to January 2022, and their lipid levels were tested from patients with consent and correlated. Student's t-test was applied to the obtained data. To convey quantitative data, measurements such as mean along with standard deviation were applied, and a p-value of 0.05 was considered statistically significant.

Results: This research included 80 subjects, 40 of whom were diagnosed with TB, and the rest (40 controls) were deemed healthy. The age group with the highest low lipid levels in pulmonary TB was 40-50 years. A chi-square test of association was conducted; this test revealed that the fraction of TB patients having lower than normal levels of total cholesterol (p = 0.0001), triglyceride level (p = 0.006), high-density lipoprotein (p = 0.009), low-density lipoprotein (p = 0.006), and body mass index (p = 0.000) was statistically significantly higher in contrast to the control group. Thus, there was a significant correlation between a higher prevalence of hypolipidemia in patients with pulmonary tuberculosis (PTB) and normal healthy individuals.

Conclusions: We observed a strong relationship between hypolipidemia and TB, indicating that patients with low lipid levels tend to have severe inflammation as compared to patients with normal lipid levels.

## Introduction

Tuberculosis (TB), caused by the organism *Mycobacterium tuberculosis*, infects the lung, afflicting it. It is a contagious disease that has always been a major healthcare problem worldwide. This disease has a high morbidity and mortality rate [1]. Nearly more than one-third of the whole population has been exposed to *M. tuberculosis *of which many live in developed and developing countries [2]. Pulmonary TB causes various pathological mechanisms involving lipid peroxidation and depletion of antioxidants. *Mycobacteria* make active forms of oxygen (reactive oxygen species [ROS]) and active nitrogen gene production (reactive nitrogen species [RNS]) by activating both mononuclear and polymorphic nuclear phagocytes [3].

Lipids have been noted to have a crucial role in the development of not only TB but also cardiovascular system (CVS) maladies [4]. A state of oxidant-antioxidant equilibrium is required in normal lung function. Inequality in the oxidant/antioxidant state can cause tissue damage and provoke an inflammation state, which contributes to the reduction of the immune system [5]. Evidence suggests that increased levels of free radicals for pulmonary TB patients have been found to reduce the body's response to antioxidant energy and contribute to the formation of lung function abnormalities [6].

There is growing evidence of a connection suggesting that low lipid levels have a direct impact on several human ailments, with TB being one among them. Cholesterol, mainly due to its involvement in heart disease, gets more attention in recent years. The density of the cell membrane is maintained by the level of cholesterol levels in our body [7-10]. This research was conducted to evaluate the correlation between lipid profile and pulmonary tuberculosis (PTB) in patients and to determine the nutritional grading of risk elements that were accompanied by hypolipidemia and TB.

## Materials and methods

An observational study was performed on TB patients attending respiratory medicine at the Saveetha Medical College and Hospital, Tamil Nadu, India, from February 2021 to January 2022. The ethical committee of Saveetha College of Allied Health Sciences approved the study with an approval number SCAHS/IRB/2021/March/031. All willing participants who were above the age of 10 years were a part of the study research. Moreover, subsequent to obtaining informed consent, 40 adult patients (male and female) recently diagnosed with PTB were selected. These patients were chosen using the following diagnostic methods: positive acid-fast bacilli (AFB) staining (two consecutive samples), GeneXpert, regular chest X-ray presenting upper extremity participation with or lacking obstruction, and with or lacking positive sputum smear but with regular PTB indications. Any patient with drug-resistant TB, visible renal, heart, neoplasm, or respiratory disease other than PTB and lung cancer, diabetes, endocrine or genetic disease, and HIV, pregnancy, or breastfeeding were all excluded from the research.

Personal data, medical history, comprising demographic details, drug history, and vitals were attained after informed consent was obtained in the language best known to them. After routine aseptic safeguards had been set, 3 ml of venous blood samples were extracted from each individual who had fasted overnight. Cholesterol, high-density lipoprotein (HDL), and low-density lipoprotein (LDL) levels were measured. Patients were classified based on their age, gender, cases, and controls. It was mandatory that blood samples be collected only after an overnight fast. Thereafter, anthropometric extents, including body weight and body mass index (BMI), were collected from the patients.

Statistical analysis

Student's t-test was applied to the obtained data. To convey quantitative data, measurements such as mean and standard deviation were applied; if the p-value was 0.05, it was determined to be statistically important. Chi-square tests were used to compare two groups for BMI, cholesterol, HDL, and LDL. A p-value < 0.05 was regarded as statistically significant. As a result, we discovered a link between PTB with BMI, cholesterol, HDL, and LDL.

## Results

The study included a total of 40 cases and 40 controls. The lipid profiles of all participants were examined. In detail, 22 males and 18 females were among the diagnosed PTB cases, while 32 males and eight females were among the controls. The majority of participants (29) were between the ages of 40 and 50. The average age was around 45 years old. Participants were divided into two groups based on their BMI: those with PTB and those without. It was noted that the mean BMI in the situation was 17.98 kg/m^2^, with a maximum of 22.23 kg/m^2^ and a minimum of 12.36 kg/m^2^, while in the control mean, the BMI was 23.65 kg/m^2^, with a maximum of 27.98 kg/m^2^ and a minimum of 15.32 kg/m^2^. In PTB, there were significantly lower lipid intensities, especially when it was in the form of total cholesterol (TC), HDL, and LDL, in contrast to the control group. In addition, concentrated lowered lipid levels in the case of PTB were established in people aged 40-50 years (Table 1).

**Table 1:** Lipid profile comparative data between cases and controls HDL: High-density lipoprotein; LDL: Low-density lipoprotein.

Lipid profile	Cases (mg/dl)	Controls (mg/dl)
Total cholesterol	119.25	186.50
Triglycerides	90.68	130.26
HDL	24.09	70.57
LDL	44.78	80.65

A chi-square test of association was conducted; this test revealed that the fraction of TB patients having lower than normal levels of total cholesterol (p = 0.0001), triglycerides (p = 0.006), HDL (p = 0.009), LDL (p = 0.006), and BMI (p = 0.000) was statistically significantly higher than the control group (Table 2). Consequently, a significant correlation in terms of the advanced pervasiveness of hypolipidemia in relation to patients with PTB and normal healthy individuals has been established.

**Table 2:** Comparative data of diverse variables between the two groups HDL: High-density lipoprotein; LDL: Low-density lipoprotein.

Variables		Case (40)	Control (40)	p-values
Body mass index	18–25	37	35	0.001
	>25	3	5	
Total cholesterol (mg/dl)	<130	29	8	0.006
	130–220	11	32	
Triglycerides (mg/dl)	<130	23	5	0.005
	130–150	17	35	
HDL (mg/dl)	<30	32	35	0.009
	>30	8	5	
LDL (mg/dl)	<100	32	35	0.006
	>100	8	5	

## Discussion

*M. tuberculosis* causes PTB, which is a chronic inflammatory illness of the lungs. Through the immune system, lipids and their metabolites have a beneficial influence on TB resistance. Lipids have been studied extensively in a variety of illness states, particularly cardiovascular and diabetes mellitus. However, knowledge is scarce about how lipids assist the immune system to fight and resist infections. Subsequently, the outcomes from this research could be used to assess the configuration of lipid profiles in terms of the pervasiveness of PTB [11].

Pérez-Guzmán et al. suggested that hypocholesterolemia was established as a risk feature in the case of PTB progression [12]. They analyzed the blood lipid profiles of 25 PTB patients and 44 household contacts. Total cholesterol, LDL cholesterol, and triglyceride levels augmented with time in people who had been in contact; however, this was not the case in patients with PTB, wherein there were statistically significant variances in regression lines (age versus lipid level) [12]. According to a multiple linear regression study, being a household advocate was linked with advanced levels of total cholesterol, LDL, HDL, and triglycerides. They concluded that the lipid profiles of PTB patients and their household contacts differed, implying that low cholesterol levels may be a risk factor for the disease. The aforementioned research and ours are comparable in that they both examined the relationship between lipid prole and PTB.

Pérez-Guzmán et al. stated that hypocholesterolemia was shown to be frequent in people having TB, being directly linked to mortality in the case of having miliary [12]. Malnutrition is known to increase the risk of TB [8,9]. Lower leptin content, which is a pleiotropic hormone, noted to be released by the adipose tissue, has been linked to being underweight (BMI: 18.5) and malnourished. Leptin has a key part in cell-mediated resistance as it promotes lymphopoiesis and increases the levels of interferon-γ (IFN-γ), a critical cytokine that aids in the control of *M. tuberculosis* infection [10]. As a result, lower levels of leptin may predispose the patient to a more severe illness by lowering IFN levels. Leptin is at reduced levels in patients having active TB. It has been noted that a diet having cholesterol-rich food speeds up the sterilizing ratio of sputum cultures in patients having PTB. According to their findings, serum TC, HDL, and LDL concentrations are lower in individuals with PTB than in healthy controls. Lipids are essential factors in the body's nutritional state. Low levels of lipids, particularly cholesterol, in the body, have been linked to an increased susceptibility to diseases such as TB. Cholesterol makes up a significant amount of the lipid content of cell membranes. The fluidity of the membrane must be maintained. Macrophages, too, require cholesterol for phagocytic functions such as cell motility, exocytosis, and endocytosis. Its phagocytic function is disrupted in cholesterol deprivation [11,13-20].

The limitations of this study were the small sample size and single-center data. The study also had a few other limitations as we did not consider the effect of other medical conditions, lifestyle factors, medications or treatments, dietary habits, or physical activity on lipid profiles, thus limiting the ability to draw meaningful conclusions about the differences between the two groups. It is imperative that large-scale studies assessing the lipid profiles of TB patients are needed to formulate new guidelines for better patient care.

## Conclusions

We found a strong statistical relationship between hypolipidemia and TB, indicating that patients with low lipid levels have more severe inflammation than patients with normal lipid levels because cholesterol is required for proper immune system function. Lipid levels should be corrected while treating TB for a better response to treatment.
